# Methods and Results in Surgery of the Stomach and Intestines*Read before Lake Keuka Medical and Surgical Association, Keuka Lake, New York, July 13, 1916.

**Published:** 1916-09

**Authors:** George W. Crile

**Affiliations:** Cleveland


### BUFFALO MEDICAL JOURNAL

Yearly Volume 72 SEPTEMBER, 1916 Number 2

### ORIGINAL ARTICLES

The right is reserved to decline papers not dealing with practical medical and surgical subjects, and such as might offend or fail to interest readers. Contributors are solely responsible for opinions, methods of expression and revision of proof.

### Methods and Results in Surgery of the Stomach and Intestines*

GEORGE W. CRILE, F. A. C. S., Cleveland.

Although during the last two decades the accuracy of the diagnosis of stomach and intestinal lesions has been greatly advanced and the mechanical technique of operations on these parts has reached a high degree of perfection, yet even in the best clinics the mortality of resections of the stomach and intestines has been reduced but slightly. Reflecting on this fact a few years ago, and being dissatisfied with my own results I concluded that further progress could be made only through a fundamental change in our conception of the problem presented by these cases.

Laboratory and clinical researches were undertaken therefore which soon disclosed certain facts which seemed to point the way to a far greater control of the fate of these patients.

As a result a practical method was evolved which has now been employed by my associate Dr. W. E. Lower and myself in 132 planned operations for lesions of the stomach and duodenum.

A study of the case histories of patients operated upon by older methods shows the principal cause of the former high mortality was not hemorrhage, not infection, not pneumonia, not a vicious circle—though each of these factors, especially infection, took a certain toll; but the principal cause of death was another insidious factor, which when once recognized became extremely obvious.

Let us first consider the clinical picture of a case with

*Read before Lake Keuka Medical and Surgical Association, Keuka Lake, New York, July 13, 1916.

pyloric obstruction. presenting the typical symptoms of the first stages of final dissolution. The face is grave, the manner dejected, the ambition lost, the complexion sallow, the pulse soft, the extremities cold, the tongue furred, the temperature normal or less than normal, the respiration somewhat accelerated, the urine scant and high colored, and the telltale lips strikingly red. The slightest exertion causes exhaustion, and—of more significance—causes respiratory distress. The patient comes to the surgeon not of his own volition—he has no will for anything but wishes to be left alone. He comes because he has been advised by his physician to make one supreme effort to turn from the threshold of the tomb.

The history of one of my own cases illustrates well the course and the fate of a patient presenting such a picture as this. In this case, the lesion was a small but obstructing pyloric cancer. The patient was borne to the hospital on a stretcher, and a large transfusion of blood was given, as a result of which the face became full and pink, the pulse strong. and the outlook for operation seemed bright. Gastroenterostomy and pylorectomy were performed. At the close of the operation all seemed well, yet the patient —without hemorrhage, without infection, without pneumonia, apparently without cause as it seemed to me then—gradually and continuously failed until his death on the second day. Literally 'a successful operation, but the patient died.'

Now the outstanding features of this case were a steady inexorable loss of muscular and mental power, an unexplained increase in the depth and the rate of respiration, pink lips, paradoxically pink lips, and acetone breath. What surgeon has not learned to fear the outcome for the patient with increased respiration and pink lips? What surgeon but hesitates to give inhalation anesthesia to a patient with increased respiration and a subnormal temperature?

The cause of the death of my patient and of many like him was acidosis. His reserve alkalinity had been virtually expended before the operation. The acid-producing inhalation anesthetic, and the extensive operation without local nerve blocking used up his entire reserve alkalinity and at the close of the operation the patient was potentially dead.

On what basis is this statement made? Why was the respiration increased, the temperature low. the brain so powerless, the lips so red? First of all, life is projected in an alkaline medium; and life ends when alkalinity is overcome. All the tissues and fluids of the body except urine, which is an excretion, are alkaline, and when the body fluids become neutral or acid, death automatically follows. The maintenance of the body temperature, muscular action.

physical injury with or without inhalation anesthesia, emotion—every process of life—is accompanied by the formation of acid by-products. Under normal conditions these constantly formed acid by-products are neutralized and the normal alkalinity of the body is maintained by the alkalies and bases normally stored in the body, and by the regulative action of the respiratory system, the liver and the kidneys.

As one would expect, the main sources of the vast potential alkalinity of the body must be the alkalies and bases derived from food. When a patient is starved by vomiting, therefore, his reserve alkalinity steadily falls until even the relatively slight acid-production resulting from his diminished activities cannot be neutralized.

As the acidity rises the respiratory rate is increased in order that the carbonic acid gas may be eliminated. The power of the blood to carry oxygen decreases simultaneously with the increased intake of oxygen consequent upon the increased respiration, hence the pink lips and reddened mucous membranes—signs of the increased amount of free oxygen in the blood. This condition is known as pink asphyxia and may be produced experimentally by giving an animal hydrochloric acid intravenously.

The intense thirst manifested by these patients is another obvious sign of diminished alkalinity as is the mounting temperature. The experienced clinician understands that this last sign indicates the last critical stage before death. The physical chemist knows that it means that the available stores of alkalies and bases have become exhausted and that the acids are therefore combining with the ammonia of the living protein molecules, the destruction of which produces the fever.

If the real hazard in these cases, is a depleted reserve alkalinity, as these premises would indicate, then the obvious problems are first—to ascertain the factors in the operation which make a further drain upon the reserve alkalinity, and second—to discover means by which to increase the reserve alkalinity.

It is obvious that no methods will avail in those cases in which the living molecules are beginning to break down. For the starved case with ruby lips, that pits on pressure, and above all, that has a rising temperature—resurrection, not operation is indicated.

All cases should have food and water. and glucose and sodium bicarbonate pushed before operation. Those cases which are nearing their ultimate acidity may be rescued by the method of von Eiselsberg; that is, by bringing up a loop of the jejunum under local anesthesia and feeding through it.

As we have stated already, surgical trauma diminishes reserve alkalinity. Therefore, in all but unquestionably good risks, in which the diagnosis is clear, a two stage operation is indicated. First a gastro-jejunostomy, resection being deferred until the functional balance is restored and the basic factor of safety is increased — usually about ten days. Thus the surgical injury is not only divided but the second and major portion occurs under improved conditions.

In our clinic each stage is performed under the technique of anociation, that the acid by-products incident to surgical trauma may be reduced to a minimum. Nitrous oxid is given in preference to ether, since we have found that nitrous oxid measurably protects the brain, the adrenals and the liver against the damaging effects of trauma of the operation, while ether not only does not protect but of itself produces further damage.

In both the first and the second stage, the patient is treated as if acidosis were expected. Saline infusion is given subcutaneously; sodium bicarbonate and glucose are given by the Murphy drip; and the patient's comfort is enhanced by the innumerable arts of a hospital force which is trained to consider the patient's welfare before all else, and to relegate to the background such rules, regulations and red tape as interfere with the patient's progress.

Sleep is induced by every possible means, for here again laboratory investigations have shown that the lesions caused by acidosis can be repaired only during sleep. It should be noted, however, that morphin is contraindicated in cases in which acidosis is present or threatened, since morphin while it prevents the further production of acids, also inhibits the neutralizing power of the body and delays or prevents the return of the body fluids to their normal alkalinity. Bromides per rectum are therefore substituted for morphin in these cases.

Aside from the protection afforded by the two-stage operation to patients in whom acidosis threatens, a striking advantage is found in cases of doubtful differentiation between cancer and ulcer.

Some years ago in a second operation upon a patient in whom a pyloric tumor had been diagnosed as cancer before and during the previous operation, six weeks before, I was astonished to find that the tumor had disappeared. Since then I have encountered three other similar cases, but in each of which, at the second operation, diagnosed as cancers before and during the first operation, one ten, one sixteen, and one twenty-one days later, the tumor had disappeared. Willy Meyer and Lilienthal have mentioned similar cases. Had I depended on appearances, and planned to operate in one seance, I should have made resections for cancer in each instance, and the end-results would have been uncertain, as in each case the patients were emaciated and starved.

The technique of the first operation,—the gastro-jejunostomy—requires no special discussion. For the resection at the second operation the old suture line is opened and if the anociation of the first stage was complete the operative field will be well mobilized and protected against infection.

In other words the first stage and the interval of adjustment accomplishes for these cases what the delay of a week accomplishes for fractures of the patella; what iodoform gauze packing accomplishes in the first stage of a laryngectomy, what acute salpingitis does for later salpingectomy. In each instance the reserves against infection are called out into the local field, and an immunity against infection in the later operation is thus guaranteed.

The technique of the resection need not be described in detail as it depends upon the number of adhesions and the location and extent of the cancerous growth. One point may be noted, however, the stomach is divided with the thermo cautery between Payr clamps and the margins of the division are sterilized by the cautery. The object of this searing is not only to secure an aseptic division of the stomach but also to coagulate the blood vessels and prevent oozing. Nor is that all—one of the commonest dangers in cancer is that of cancer implantation on the cut ends of the tissues divided in the operation. The searing makes a dry charred surface which cannot sustain the growth of cancer tissue. To prevent leakage the divided ends are inverted and closed with the shoemaker stitch, and as a further protection a series of interrupted silk sutures reinforce the shoemaker stitch which is of chromic gut.

**Summary**

1. In cases in which resection of the stomach is indicated the reserve alkalinity is usually lessened, often reduced to a minimum.

2. In these cases, such measures should be employed as will obviate the further depletion of the body's stores of alkalies and bases, and the reserve alkalinity should be increased.

3. Because of its conserving action, and its pleasing subjective effect upon the patient nitrous oxid is the anesthetic of choice.

4. The acid producing effects of operative trauma should be reduced to a minimum by employing the technique of anociation.

5. In resection the operation should be performed in two stages—first, gastrojejunostomy, second, resection after nutritional balance is restored.

6. Water and subcutaneous saline infusions, glucose and soda bicarbonate per rectum, increase the reserve alkalinity and consequently conserve the patient.

7. Bromides per rectum conserve the patient by diminishing the transformation of energy, thus diminishing acid production; and by inducing sleep, the only means by which the damage done to the energy transforming organs by excessive acid by-products can be repaired.

8. The division of the stomach by the cautery and the thorough searing of the cut edges with moderate heat sterilizes against pyogenic infections and against cancer growth and prevents bleeding—three of the most important considerations in gastric resection.

1021 Prospect Avenue.

---

**Railways and National Defense.** W. L. Park, V. P. Ill. Cent. R. R., Railway Surg. Jour., July, points out that the limited amount of double trackage in the U. S. would be a serious impediment. A 14 inch gun requires a 100-ton flat car, being 48 ft. long and 7 ft. wide and weighing 139,000 pounds. The new 16 inch gun is 67 ft. long, over 7 ft. wide and weighs 367,000 pounds. There are few flat cars of the proper size available in the U. S. The War Dept. estimates the requirements of an infantry regiment at 5 sleepers, 43 coaches, 5 baggage cars, 15 box cars, 9 stock cars, 8 flat cars, occupying 5,150 ft. of track. A cavalry regiment would require 8 sleepers, 28 coaches, 8 baggage cars, 25 box cars, 72 stock cars, 9 flat cars, occupying 7,850 ft. of track. An infantry division would require 1507 cars of various kinds, occupying about 15 miles of track; a cavalry division, 1,302 cars, 14 miles plus. A locomotive and tender must be allowed for about every 17 cars. He advocates a board of army experts co-operating with practical railroad men.

---

**Test for Occult Blood in the Stool.** Wagner (Arch. fur Verdauungskrankh., Vol. XX., No. 4). A little of the stool (if formed, from its central portion) is spread over a clean slide. Over this the reagent is poured, a bit of benzidin, dissolved in 2 c.c. glacial acetic acid, plus 20 drops of 3 per cent. hydrogen peroxide. A blue color shows the presence of blood. The test is as delicate and reliable as any benzidin test. Extraneous ferments, which constitute a possible source of error, may be excluded by heating the film.

